# Household air pollution exposure in adult women is associated with increased carotid intima-media thickness: A cross-sectional study of the Household Air Pollution Intervention Network trial

**DOI:** 10.1016/j.ijheh.2025.114649

**Published:** 2025-09-01

**Authors:** Lindsay J. Underhill, Lisa de las Fuentes, Laura Nicolaou, Shakir Hossen, Anaite Diaz-Artiga, Ajay Pillarisetti, Aris T. Papageorghiou, Florien Ndagijimana, Ghislaine Rosa, Gurusamy Thangavel, John P. McCracken, Kalpana Balakrishnan, Krishnendu Mukhopadhyay, Kyle Steenland, Lisa M. Thompson, Lance A. Waller, Maggie L. Clark, Michael A. Johnson, Sarada Garg, Sankar Sambandam, Suzanne M. Simkovich, Vigneswari Aravindalochanan, Kendra N. Williams, Wenlu Ye, Jennifer L. Peel, Thomas F. Clasen, William Checkley, Victor G. Davila-Roman, for the HAPIN Investigators

**Affiliations:** a Cardiovascular Division and Global Health Center, Washington University School of Medicine, St. Louis, MO, USA; b Center for Biostatistics and Data Science, Institute for Informatics, Data Science, and Biostatistics (I2BD), Washington University School of Medicine, St. Louis, MO, USA; c Division of Pulmonary and Critical Care, School of Medicine and Center for Global Non-Communicable Disease Research and Training, Johns Hopkins University, Baltimore, MD, USA; d Department of Preventive Medicine, University of Mississippi Medical Center, Jackson, Mississippi, USA; e Center for Health Studies, Universidad del Valle de Guatemala, Guatemala City, Guatemala; f Division of Environmental Health Sciences, University of California at Berkeley, Berkeley, California, USA; g Nuffield Department of Women’s and Reproductive Health, University of Oxford, Oxford, UK; h Eagle Research Centre, Kigali, Rwanda; i Department of Infectious and Tropical Diseases, London School of Hygiene & Tropical Medicine, London, UK; j Department of Environmental Health Engineering, Sri Ramachandra Institute for Higher Education and Research, Chennai, India; k Gangarosa Department of Environmental Health, Rollins School of Public Health, Emory University, Atlanta, Georgia, USA; l Nell Hodgson Woodruff School of Nursing, Emory University, Atlanta, GA, USA; m Department of Biostatistics and Bioinformatics, Rollins School of Public Health, Emory University, Atlanta, Georgia, USA; n Department of Environmental Health Sciences, University of Georgia, Athens, GA, USA; o Department of Environmental and Radiological Health Sciences, Colorado State University, Fort Collins, Colorado, USA; p Berkeley Air Monitoring Group, Berkeley, CA, USA; q Division of Healthcare Delivery Research, MedStar Health Research Institute, Hyattsville, MD, USA; r Division of Pulmonary and Critical Care Medicine, Georgetown University, Washington, DC, USA; s World Health Organization, Geneva, Switzerland; t Global Health Innovation Network, WashU School of Public Health, St. Louis, MO, USA

**Keywords:** Household air pollution, Atherosclerosis, Carotid artery ultrasound

## Abstract

**Background:**

Cardiovascular disease is the leading cause of global morbidity and mortality, disproportionately affecting people in low- and middle-income countries (LMICs). Biomass fuels used for cooking in LMICs contribute significantly to household air pollution (HAP), which has been associated with inflammation, oxidative stress, and other pathways linked to atherosclerosis. We evaluate the association between HAP exposure and atherosclerosis by use of carotid artery ultrasound.

**Methods:**

An exposure-response analysis was conducted using cross-sectional baseline data from 397 women aged 40–79 years from the Household Air Pollution Intervention Network (HAPIN) trial in Guatemala, India, Peru, and Rwanda. Participants underwent ultrasound evaluation of their carotid arteries to measure intima-media thickness (CIMT) and atherosclerotic plaques. Additionally, 24-h personal exposures to particulate matter (PM_2.5_), carbon monoxide (CO), and black carbon (BC) were assessed.

**Findings:**

Mean 24-h PM_2.5_ exposure was 119 μg/m^3^ (range 10–803), BC was 13 μg/m^3^ (range 1.1–72), and CO was 2.3 ppm (range 0–39). Mean and maximal unadjusted CIMT measurements were 0.64 ± 0.13 mm and 0.75 ± 0.14 mm, respectively. Prevalence of atherosclerotic plaques was 7.1 % (range: 0.8 %–11.6 % by country). In adjusted linear models, each 10 μg/m^3^ increase in PM_2.5_ was associated with a 0.001 mm increase in mean CIMT (95 % CI: 0 to 0.002) and a 0.002 mm increase in maximal CIMT (95 % CI: 0 to 0.003). For CO, each 10 ppm increase was associated with a 0.04 mm increase in maximal CIMT (95 % CI: 0 to 0.08), with the highest quartile of CO exposure having 0.036 mm and 0.05 mm greater mean and maximal CIMT (95 % CI: 0.002 to 0.07; 0.01, 0.09), respectively, than the lowest quartile. No significant associations were found between BC and CIMT or between any exposures and carotid atherosclerotic plaque.

**Interpretation:**

In this cross-sectional study, higher personal exposures to PM_2.5_ and CO were associated with greater mean and maximal CIMT, a well-established biomarker of atherosclerosis, further supporting the association between HAP and cardiovascular disease.

## 1. Background

Cardiovascular disease is the leading cause of global morbidity and mortality, disproportionately affecting people living in low- and middle-income countries (LMICs). A major underlying cause of CVD is atherosclerosis, a process characterized by the accumulation of fatty and fibrous material in the innermost layer of the arterial wall (the intima) (Libby, 2019). This accumulation results from long-term exposure to risk factors such as high cholesterol, atherogenic lipoproteins, and inflammatory cytokines, as well as traditional cardiometabolic risk factors like diabetes, hypertension, and obesity. Environmental risk factors such as air pollution have also been associated with cardiovascular events such as myocardial infarction, heart failure, cardiac arrhythmias, and stroke. Household air pollution (HAP), generated by the burning of biomass, other solid fuels, and kerosene for cooking, heating, and lighting, affects an estimated 3.6 billion people worldwide and has also been associated with CVD (IHME). HAP exposure is high in LMICs, where inefficient stoves and inadequate ventilation lead to the indoor accumulation of HAP pollutants, such as fine particulate matter (PM_2.5_), carbon monoxide (CO), and black carbon (BC). These pollutants may drive systemic inflammation and oxidative stress, key mechanisms in the development of atherosclerosis and CVD. Despite the significant burden of HAP exposure in LMICs, its association with CVD remains poorly studied and thus represents an important public health gap.

Carotid intima-media thickness (CIMT) is an ultrasound measure of carotid arterial wall thickness and a well-established imaging biomarker of atherosclerosis. Increased CIMT is a strong predictor of CVD risk, including myocardial infarction and stroke (Willeit et al., 2020). Studies from our group and others have shown an association between increased CIMT and chronic exposure to both HAP and ambient air pollution (Ofori et al., 2018; Ranzani et al., 2019; Painschab et al., 2013; Provost et al., 2015). Several studies have observed higher CIMT among women who self-reported HAP exposure from wood-burning cookstoves in Nigeria, South India, and Peru (Ofori et al., 2018; Ranzani et al., 2019; Painschab et al., 2013). For ambient air pollution, associations have been documented primarily at low-to-medium pollution levels in high-income countries. For example, exposure-response analyses have demonstrated that exposure to ambient and traffic-related PM_2.5_ is positively associated with CIMT, although exposure measurements were ecological, based on local area sampling, or on modeled exposures (Ranzani et al., 2019; Liu et al., 2015; Sommar et al., 2022).

While an association between HAP and CIMT has been previously demonstrated, the specific exposure-response relationship measured by personal HAP monitors and CVD biomarkers, particularly CIMT and atherosclerotic plaque, has not been well-characterized. The current study aims to address this gap by measuring the association of CIMT and plaque presence with 24-h personal exposures to PM_2.5_, BC and CO from 397 women aged 40–79 years in Guatemala, India, Peru, and Rwanda participating in the Household Air Pollution Intervention Network (HAPIN) trial. The hypothesis of the study is that higher personal exposures will be associated with thicker CIMT and carotid artery atherosclerotic plaques.

## 2. Methods

### 2.1. Study design

The current analysis uses baseline data collected during the HAPIN trial, a multi-country randomized controlled trial (RCT) of a clean cooking intervention in rural communities in Guatemala, India, Peru, and Rwanda. HAPIN enrolled 3200 households (800 per country) with pregnant women (18–34 years old at 9–19 weeks gestation) who exclusively or primarily used biomass fuels for cooking. An additional 418 older adult women (40–79 years old) residing in the same household were also enrolled and constitute the population for the current analysis. Households were randomized 1:1 to receive either the HAPIN intervention package—including an LPG stove, continuous fuel supply, and behavioral messaging to promote exclusive LPG use—or to continue using biomass fuels. Prior to randomization and intervention delivery, baseline data was collected via sociodemographic and health history surveys, 24-h personal exposure monitoring for PM_2.5_, BC, and CO was collected, and clinical health assessments were conducted, including carotid artery ultrasound imaging for CIMT and plaque identification. Full trial details and protocol has been described previously (Clasen et al., 2020).

The study protocol was approved by all participating institutional review boards (IRBs) and/or ethics committees from Emory University (00089799), Johns Hopkins University (00007403), Washington University in St. Louis (WashU) (201611159), Sri Ramachandra Institute of Higher Education and Research (IEC-N1/16/JUL/54/49), the Indian Council of Medical Research—Health Ministry Screening Committee (5/8/4–30/(Env)/Indo-US/2016-NCD-I), Universidad del Valle de Guatemala (146–08-2016), Guatemalan Ministry of Health National Ethics Committee (11–2016), Asociación Benefica PRISMA (CE2981.17), the London School of Hygiene and Tropical Medicine (11664–3), and the Rwandan National Ethics Committee (No.016/RNEC/2018).

### 2.2. Carotid artery ultrasound methods

Carotid artery ultrasound imaging was performed by certified sonographers using a portable ultrasound system (SonoSite Edge, Fujifilm-SonoSite Inc, Bothell, WA, USA) with a 7–15 MHz linear transducer. Ultrasound system settings, transducer placement, and other details have been described previously (Dávila-Román et al., 2021). Briefly, ECG-gated video and still frame images of the posterior wall of the distal 1 cm of the right and left carotid arteries of the common carotid artery (just proximal to the carotid artery bulb) were obtained as previously described (Stein et al., 2009). After identification of the 1-cm region of interest, the semi-automated edge detection software algorithm provided 100 separate dimensional measurements. From these 100 measurements, CIMT values were derived: mean CIMT (average thickness of the artery intima-media layer across the 100 measurements) and maximal CIMT (highest thickness value observed in the segment). Atherosclerotic plaques were defined as a focal intima-media thickness >1.5 mm or focal wall thickening that protruded into the lumen >0.5 mm or >50 % of the surrounding common carotid, bulb and internal carotid segments (Stein et al., 2009; Lin et al., 2018). Complete studies included approximately 8 images. All images were obtained by trained sonographers (see below) with acquisition taking approximately 20–30 min. Case report forms were completed online by site sonographers and linked to each ultrasound study. Site sonographers (three per site) underwent a 2-week in-person standardized training workshop at WashU.

Completed studies were uploaded to a cloud-based image server (Trice Imaging Inc., Del Mar, CA, USA) by site sonographers for subsequent offline analysis at the Ultrasound Core Laboratory (UCL) at WashU. For CIMT, automatic edge detected values for the mean and maximal CIMT are presented as the average of three each of the right and left common carotid arteries. Uploaded studies were reviewed by UCL sonographers blinded to study site and subject identity to assess image quality and adequacy of measurements (Dávila-Román et al., 2021).

### 2.3. Household air pollution personal exposure assessment

The HAPIN exposure assessment protocol has been described in detail previously (Johnson et al., 2020). Prior to randomization and intervention delivery, participants wore lightweight monitors for 24-h to measure their baseline personal exposure to PM_2.5_, BC, and CO. Participants carried the monitors in custom-designed aprons or vests that were developed in collaboration with participants to ensure appropriateness and comfort. During activities such as bathing and sleeping, participants were instructed to hang the aprons in a safe location nearby. Although limited to a single 24-hr period, this approach was designed to capture total indoor and outdoor exposure during participants’ routine daily activities. This can provide a more precise individual-level estimate than modeled or measured area-based measurements that are prone to exposure misclassification.

PM_2.5_ and BC exposures were measured using the Enhanced Children’s MicroPEM (ECM), a validated, lightweight particulate sampler (RTI Inc, Research Triangle Park, NC, USA). The ECM uses a built-in internal 0.3 L/min pump to draw air through an impactor attached to a cassette containing 15-mm Teflon^®^ filters (PT15-AN-PF02; MTL Corporation, Minneapolis, MN), onto which particulates deposit. Gravimetric measurements of PM_2.5_ mass concentration were based on the pre- and post-weights of the filters, adjusted by sampling time and flow rate. Real-time, continuous PM_2.5_ exposure was collected by an internal light-scattering sensor. The ECM also monitored temperature, relative humidity, and filter-pressure drop. When a gravimetric sample was deemed invalid, due to missing or damaged filters or flow faults, nephelometer data were used to estimate PM_2.5_ concentrations. BC was estimated post-sampling on the ECM filters using the SootScan Model OT-21 Optical Transmissometer (Magee Scientific, Berkeley, CA, USA). Real-time, continuous monitoring of CO was conducted using the Lascar EL-USB-300 monitor (Lascar Electronics, Erie, PA), a lightweight, data-logging monitor. The Lascar instrument runs on batteries, has a sensing range between 0 and 300 ppm, and uses an electrochemical cell to measure CO.

### 2.4. Anthropometric measurements and surveys

Body Mass Index (BMI) was calculated for each participant, using the formula of weight in kilograms divided by the height in meters squared (kg/m^2^). Blood pressure (BP) was measured in triplicate on the right arm using an automatic digital BP monitor (OMRON HEM907XL) as detailed in the study protocol (Clasen et al., 2020). Surveys were administered to participants by trained field staff proficient in the local language to determine demographic and socioeconomic status (SES), health history, household characteristics, exposure to environmental tobacco, and other characteristics. SES was determined using an index constructed through principal components analysis (PCA) based on household assets, water and sanitation quality, food insecurity, education level, and housing materials, as described in detail previously (Nicolaou et al., 2022). To help ensure the validity and reliability of the collected data, all questionnaires were piloted prior to implementation.

### 2.5. Statistical methods

Single- and multi-variable linear regression models were run to test the association between personal exposures (PM_2.5_, BC or CO) and mean CIMT, maximal CIMT, and atherosclerotic plaque. Covariates were selected *a priori* using a directed acyclic graph (DAG) to identify a minimally sufficient set of factors for adjustment, to address potential confounders, and to ensure chosen covariates did not function as colliders (Fig. S1). The final models included the following covariates: study site (Guatemala, India, Peru, Rwanda), SES index (continuous: higher is worse), secondhand smoke (categorical: yes or no), and age (continuous). Separate models for PM_2.5_, BC, and CO were run due to moderate-to-high correlations between pollutants (PM_2.5_ and BC: Spearman’s *ρ* = 0.49; PM_2.5_ and CO: Spearman’s *ρ* = 0.52; CO and BC: Spearman’s *ρ* = 0.53) (Fig. S2).

Linear and log-linear models were tested, where pollutant exposures (PM_2.5_, BC, CO) were natural log-transformed (Fig. S3). To assess potential non-linear exposure-response relationships, exposures were categorized into quartiles (Fig. S4). Non-linear associations were further examined using generalized additive models (GAMs) with smoothing splines and tensor-product smooths (Fig. S5). Model performance and fit was evaluated using visualizations and Akaike Information Criterion (AIC), where lower AIC values indicate a better fit (Figs. S3–S5).

## 3. Results

### 3.1. Baseline characteristics of the study population

Of the 418 older women enrolled in HAPIN, 21 were missing baseline CIMT measurements and excluded from the current analysis. Accordingly, the study population consisted of 397 older adult women with CIMT measurements across the four study sites (Table 1). The mean age (52 ± 8 years) was similar across countries. BMI was highest in Peru and lowest in India. Second-hand smoke exposure (self-reported) was highest in India (28 %) followed by Rwanda (7 %) and Guatemala (5 %), with no secondhand smoking reported in Peru. Only 12 % and <1 % of participants in Rwanda and Peru, respectively, reported smoking previously; no participants reported smoking currently. Reported clinical history of high blood pressure (5.6 %), high cholesterol (1.2 %), and other heart disease (1.9 %) was low overall. More than half (57 %) were employed outside the household. The SES index was highest (indicating worse off) in Rwanda, followed by Guatemala, Peru, and India.

### 3.2. Distribution of personal HAP exposures

Twenty-four-hour personal exposure measurements were available as follows: PM_2.5_: 91 % (362 participants); BC: 79 % (313 participants) and CO: 83 % (329 participants) (Table 2; Fig. S6). Across sites, the median 24-h personal exposure to PM_2.5_ was 87 μg/m3 (range: 60 μg/m3 in Peru to 110.3 μg/m3 in Guatemala). Notably, 81 % of participants experienced personal PM_2.5_ exposures exceeding the World Health Organization annual interim target-1 guideline of 35 μg/m^3^. Across sites, overall median 24-h exposure to BC was 11 μg/m^3^ (range: 10 μg/m^3^ in Peru to 12 μg/m^3^ in Guatemala), and 24-h median CO exposure was 1.4 ppb (range: 0.6 ppb in Rwanda to 2.3 ppb in Peru).

### 3.3. Distribution of CIMT and carotid artery atherosclerotic plaques

The average mean and maximal CIMT were 0.64 ± 0.13 mm and 0.75 ± 0.14 mm, respectively (Table 3). Both mean and maximal CIMT were highest in Rwanda, followed by Guatemala, India, and Peru. The overall prevalence of atherosclerotic plaques was 7.1 %, with the highest prevalence in Guatemala (11.6 %) and lowest in Peru (<1 %). Overall, mean and maximal CIMT were positively correlated with age (Spearman’s ρ = 0.45, p < 0.001 and ρ = 0.42, p < 0.001, respectively) and SBP (ρ = 0.44, p < 0.001 and ρ = 0.46, p < 0.001, respectively) (Fig. S7).

### 3.4. Associations between personal exposure to HAP pollutants and CIMT and carotid artery atherosclerotic plaques

Statistical models assessing the relationship between 24-h personal exposures and CIMT or atherosclerotic plaque were restricted to participants who had both relevant exposure and outcome data and included adjustments for the *a priori* covariates country, age, secondhand smoke exposure, and SES. In adjusted linear models, each 10 μg/m^3^ increase in PM_2.5_ was associated with a 0.001 mm increase in mean CIMT (p = 0.008; 95 % CI: 0 to 0.002) and a 0.002 mm increase in maximal CIMT (p = 0.005; 95 % CI: 0 to 0.003) (Table 4). For CO, each 10 ppm increase was associated with a 0.04 mm increase in maximal CIMT (p = 0.48; 95 % CI: 0 to 0.08). Additionally, on average, those in the fourth quartile of CO exposure had a 0.036 mm greater mean CIMT (95 % CI: 0.002, 0.071; p = 0.04) and 0.05 mm greater maximal CIMT (p = 0.02; 95 % CI: 0.01 to 0.09) compared to those in the first quartile. The association between CO and mean CIMT was positive but did not reach statistical significance at the α = 0.05 level. No significant exposure-response relationships were observed between black carbon and mean or maximal CIMT (Table 4) or between atherosclerotic plaques and any pollutant exposures (Table S1).

## 4. Discussion

In this cross-sectional study of older adult women living in households that primarily used biomass fuel for cooking in four LMICs, 24-h personal exposures to PM_2.5_, BC, and CO and carotid artery ultrasound (mean CIMT, maximal CIMT and atherosclerotic plaques) were measured. Higher personal exposure to PM_2.5_ was associated with greater mean and maximal CIMT, and higher CO exposure was associated with maximal CIMT. Additionally, women in the highest quartile of CO exposure had significantly greater maximal CIMT compared to those in the lowest quartile. To our knowledge, this is the first multi-country study to measure associations between directly measured personal exposures to PM_2.5_, BC and CO among women chronically exposed to household biomass smoke and CIMT, a well-validated biomarker of atherosclerosis and CVD (Ruijter et al., 2012; Lorenz et al., 2007, 2012; van den Oord, 2013; O’Leary et al., 1999). These findings address a critical gap in understanding the potential impacts of HAP exposure, which is widespread globally and particularly in LMICs, on the development of CVD, the most common cause of death and disability worldwide.

The current study contributes to the limited existing literature on HAP and atherosclerosis. Previous studies have primarily relied on indirect proxies of HAP exposure, such as self-reported biomass stove use or geographic location. For example, an earlier study in Peru from our group reported that rural participants (who primarily used biomass fuels) exhibited significantly thicker mean and maximal CIMT and had a higher prevalence of atherosclerotic plaque compared to urban women (Painschab et al., 2013). Similarly, a study of rural dwelling women in southern Nigeria found that biomass fuel users had increased CIMT compared to clean fuel users (Ofori et al., 2018). Another study in peri-urban southern India found a positive but non-significant association between predicted personal PM_2.5_ exposure and CIMT (Ranzani et al., 2020). Notably, the average PM_2.5_ exposure (61 μg/m^3^, IQR = 12.9) was approximately half of that observed in the present study. Furthermore, predicted personal exposures derived from modeling a subset of measurements were used in the Ranzani et al. study, rather than direct personal monitoring. Differences in self-reported biomass use (60 % in their population) may further explain differences in findings. Additionally, a study of adults (40–79 years old) from China reported a borderline significant positive association between directly measured personal PM_2.5_ exposure and CIMT. Together, these studies suggest a probable association between HAP exposure and vascular thickening; however, most relied on proxy measures of HAP exposure. The present study strengthens this evidence base by using direct 24-h personal exposure measurements across diverse LMIC settings and demonstrating significant positive associations between CIMT and both PM_2.5_ and CO, suggesting a potential pollutant-specific relationship.

Our finding that PM_2.5_ is positively associated with CIMT is biologically plausible as PM_2.5_ can induce systemic inflammation and oxidative stress, two key pathways in the development of the vascular thickening (measured by increased CIMT) that precedes atherosclerosis. Our findings also generally align with epidemiological evidence from higher-income settings with lower exposure levels. For example, a 2015 meta-analysis of pooled data from 9 studies conducted in Europe and North America reported that each 5 μg/m^3^ increase in ambient PM_2.5_ was associated with a 12.1 μm increase in CIMT (Provost et al., 2015). Subsequent studies have generally supported a positive association between PM_2.5_ and CIMT, though with some heterogeneity across studies (Jilani et al., 2020; Kim et al., 2024). While our results are broadly consistent with this literature, direct comparisons should be made with caution. The studies from high-income countries typically relied on measured or modeled ambient exposures at much lower concentrations, whereas our study measured total 24-h personal exposure—including both indoor and outdoor sources—at substantially higher levels in rural and peri-urban LMIC settings. The smaller effect sizes observed in our study may reflect attenuation at higher exposures, non-linear exposure-response relationships, or physiological adaptation to chronic biomass smoke. Nevertheless, findings from these ambient and personal exposure studies are complementary and together contribute to a more complete understanding of the vascular impacts of air pollution.

The present study found an association between CO exposure and maximal CIMT, particularly among those participants in the highest exposure quartile. While the acute effects of CO poisoning and cardiopulmonary disease are well established (e.g., myocardial ischemia, arrhythmias, pulmonary edema, cardiac dysfunction), the effects of chronic CO exposure on cardiopulmonary disease and/or development of atherosclerosis are less well understood. Epidemiologic studies have shown associations between short-term exposure to ambient CO with CVD morbidity and mortality (Chen et al., 2007, 2011; Bell et al., 2009; Tian et al., 2015). A recent study of 272 cities in China reported strong evidence of an association between short-term exposure to ambient CO and increased CVD mortality, and particularly coronary heart disease mortality (Chen et al., 2011). CO contributes to vascular damage through mechanisms like hypoxia and oxidative stress, leading to endothelial dysfunction and atherosclerosis. A case-control study in Turkey among 40 barbecue workers with high CO exposure vs. 48 controls reported an association between higher carboxyhemoglobin levels, a biomarker of CO exposure, and increased CIMT. In the current study, a significant association between CO exposure and maximal CIMT (but not mean CIMT) was found. While mean CIMT is more widely used, maximal CIMT is considered a more sensitive biomarker of atherosclerosis progression as it captures focal thickening of the arterial wall, a precursor to atherosclerotic plaque development (Bots et al., 2003; O’Brien et al., 2024). Together, results from prior studies and our findings suggest a potential role of chronic CO exposure in CVD development.

This is the first study to measure associations between CIMT and directly measured personal exposure to BC. While a small positive association between BC and CIMT was found, it did not reach statistical significance. In contrast, Wilker et al. showed a significant association between long-term BC exposure and increased CIMT in a senior population in Massachusetts (Wilker et al., 2013). Similarly, Kim et al. found a positive association between BC and CIMT in middle-aged adults from four US cities, after adjustment for demographic, behavioral, socioeconomic, and comorbidity factors, and in subgroup analyses by race and sex (Kim et al., 2024). BC can stimulate systemic inflammation and oxidative stress pathways implicated in the development of cardiovascular disease (Kim et al., 2024; Alexeeff et al., 2011; Jiang et al., 2020). The lack of a significant association between BC and CIMT in the present study may be explained by differences in exposure assessment. Unlike previous studies, which relied on ambient air pollution or modeled BC exposures from traffic-related pollution in urban US settings, the present study used personal exposure measurements in populations primarily exposed to HAP from biomass combustion. The composition and source of PM_2.5_, including BC, may differ between traffic-related and biomass-related pollution. It is possible that other components of PM_2.5_, rather than BC, are the primary drivers of the observed associations in the present study.

Finally, a study of 606 asymptomatic low cardiovascular risk adults from Australia found that PM_2.5_ ambient air pollution was associated with the degree of coronary artery calcification, another imaging biomarker of atherosclerosis, independent of other risk factors (Huynh et al., 2020). Collectively, evidence from prior studies and ours using imaging biomarkers of vascular remodeling and atherosclerosis indicate that air pollution may play a role in residual CVD risk beyond traditional risk factors.

The present cross-sectional study did not find a significant association between 24-h personal exposure to PM_2.5_, BC, or CO and atherosclerotic plaques. Few studies in high exposure settings exist for comparison and results are mixed. Similar to our study, Kanagasabai et al., reported positive, albeit insignificant associations between total area of atherosclerotic plaques and self-reported solid fuel use and PM_2.5_ exposure (Kanagasabai et al., 2021). In contrast, in Peru, our group reported that greater self-reported use of biomass fuels for cooking was associated with a higher prevalence of atherosclerotic plaques (Painschab et al., 2013). Differences in overall atherosclerotic plaque prevalence (26 % in Painschab et al. vs. 7 % in the current study) and population characteristics (inclusion of both sexes vs. only women in the current study) may also explain the discrepant findings. In a review of high-income, low-exposure settings, Julani et al. reported mostly positive but some weak associations between PM_2.5_ and atherosclerotic plaques (Jilani et al., 2020). Conversely, a cross-sectional study in a Swedish cohort did not find an association between annual mean PM_2.5_ and atherosclerotic plaques (Hasslöf et al., 2020).

### 4.1. Limitations

The present study is cross-sectional, which limits causal inference between personal exposures and CIMT outcomes. Additionally, baseline personal exposure assessment occurred over a single 24-h period, which may not capture intra-individual variability related to seasonal or weekly routines or reflect long-term exposures patterns. However, the advantage of this approach is that it captures total exposure across all indoor and outdoor environments during the limited sampling period, thereby providing a more precise individual-level estimate than modeled or measured area-based samples that are prone to exposure misclassification. Additionally, since all participants were still exclusive or primary biomass users at the time of baseline measurement, and stove and fuel type are strong drivers of exposure, we expect historical fuel use and exposure levels to have been relatively stable and the one-time measurement to be a reasonable estimate of habitual exposure. Additional limitations include reliance on participant adherence to wearing the monitors during the 24-h exposure assessment period. To mitigate this, we piloted the monitoring protocol using context-appropriate vests and provided training to ensure comfort and compliance. Furthermore, the participant population was concentrated at the higher end of the exposure distribution, which may have limited our ability to detect associations at lower exposure levels. Finally, observed differences in exposure levels and CIMT across study sites may reflect regional clustering of fuel types, cooking practices, and underlying cardiovascular risk profiles. Despite adjustment for study site in our models, unmeasured heterogeneity of these individual factors may have reduced our ability to detect additional associations.

### 4.2. Conclusions

In this cross-sectional study of adult women living in households that primarily cook with biomass fuels, higher personal exposures to PM_2.5_ and CO were associated with greater mean and maximal CIMT, a well-established biomarker of atherosclerosis, further supporting the association between HAP and cardiovascular disease.

## Supplementary Material

Underhill_IJHEH_2025_SI

## Figures and Tables

**Table 1:** Baseline characteristics of all study participants who had CIMT measurements, by study site.

Variable	Overall	Guatemala	India	Peru	Rwanda

N	397	133	93	128	43
Age (mean yrs ± SD)	52 ± 8	54 ± 8	49 ± 7	52 ± 8	53 ± 9
Household size (mean ± SD)	6 ± 2.6	8 ± 2.9	4 ± 1.4	6 ± 2	6 ± 2
SES Index (±SD) (higher is worse)	0.2 ± 1	−0.2 ± 0.8	1 ± 0.7	1 ± 0.5	−1.2 ± 0.7
BMI (mean kg/m^2^ ± SD)	25 ± 5	26 ± 4	21 ± 3	29 ± 5	23 ± 4
SBP (mean mmHg ± SD)	116 ± 18	120 ± 20	122 ± 14	108 ± 12	119 ± 17
DBP (mean mmHg ± SD)	70 ± 11	71 ± 11	77 ± 10	64 ± 9	73 ± 9
Self-report health history:					
Hypertension (%)	6	10	0	5	9
Diabetes (%)	3	4	4	2	2
High Cholesterol (%)	1	2	0	2	0
Heart Disease (%)	2	3	0	1	5
Smoking History (%)	2	0	0	1	12
Secondhand Smoke – Current (%)	9	5	28	0	7
Hypertension medication: current (%)	2	2	0	2	7

**Table 2:** Personal 24-Hour exposures to PM_2.5_ (μg/m^3^), BC (μg/m^3^), and CO (ppm), by study site, among those with CIMT measurements.

	All	Guatemala	India	Peru	Rwanda

**PM2.5 (μg/m** ^**3**^ **)**	N = 362	N = 129	N = 82	N = 110	N = 41
Mean ± SD	119 ± 121.2	144 ± 127.9	114 (127.8)	106 (119.9)	93 (69)
Median [IQR]	87 [44, 139]	110 [71, 180]	74 [44, 125]	60 [29, 123]	75 [49, 118]
Range	10, 803.4	14, 803	10, 760	13, 698	15, 312
**BC (μg/m** ^**3**^ **)**	N = 313	N = 115	N = 79	N = 91	N = 28
Mean ± SD	13 ± 9.8	13 ± 6.3	14 ± 10.9	13 ± 13	10 ± 5
Median [IQR]	11 [7, 16]	12 [9, 16]	11 [7, 19]	10 [3, 16]	10 [7, 13]
Range	1.1, 72	1.1, 47	1.6, 69	1.3, 72	2.8, 20
**CO (ppb)**	N = 329	N = 121	N = 79	N = 91	N = 38
Mean ± SD	2.3 ± 3.5	1.8 ± 1.8	1.6 ± 3	3.9 ± 5.2	1.3 ± 1.8
Median [IQR]	1.4 [0.5, 3]	1.4 [0.6, 2.6]	0.7 [0.2, 2.1]	2.3 [1, 5.3]	0.6 [0.3, 1.6]
Range	0.001, 39	0.001, 9	0.001, 21	0.00067, 39	0.0317, 9

* Population restricted to participants with valid exposure and CIMT measurements.

**Table 3:** Mean and maximum CIMT and prevalence of carotid artery atherosclerotic plaque, by study site.

	Overall N = 397	Guatemala N = 133	India N = 93	Peru N = 128	Rwanda N = 43

**Mean CIMT (mm)**					
Mean ± SD	0.64 ± 0.13	0.69 ± 0.12	0.61 ± 0.13	0.59 ± 0.10	0.72 ± 0.13
Range	[0.4, 1.38]	[0.46, 1.04]	[0.42, 1.38]	[0.4, 0.99]	[0.47, 1.01]
**Maximum CIMT (mm)**					
Mean ± SD	0.75 ± 0.14	0.79 ± 0.14	0.73 ± 0.17	0.71 ± 0.11	0.83 ± 0.14
Range	[0.49, 1.7]	[0.52, 1.23]	[0.51, 1.7]	[0.49, 1.11]	[0.56, 1.12]
**Carotid artery atherosclerotic plaques**					
	N = 393	N = 129	N = 93	N = 128	N = 43
Prevalence (%)	7.1 %	12 %	9.7 %	0.8 %	6.7 %

**Table 4:** Associations between personal 24-h exposure to PM_2.5_ (μg/m^3^), BC (μg/m^3^), and CO (ppm) and mean and maximum CIMT (mm) across linear, log-linear, and quartile-based models.

Pollutant	Model Type	Estimate	95 % CI	p-value	AIC

*Mean CIMT* PM2.5	Linear	0.001	(0, 0.002)	**0.008** *	−595.2
	Log linear	0.026	(−0.002, 0.057)	0.075	−591.2
	Quartile 2	−0.005	(−0.036, 0.027)	0.771	−587.9
	Quartile 3	0.002	(−0.031, 0.034)	0.928	
	Quartile 4	0.024	(−0.008, 0.056)	0.144	
BC	Linear	0.003	(−0.01, 0.02)	0.668	−497.0
	Log linear	0.002	(−0.034, 0.038)	0.939	−496.8
	Quartile 2	0.012	(−0.024, 0.049)	0.499	−494.9
	Quartile 3	−0.008	(−0.044, 0.028)	0.667	
	Quartile 4	−0.009	(−0.044, 0.026)	0.612	
CO	Linear	0.03	(−0.001, 0.06)	0.140	−535.3
	Log linear	0.016	(−0.002, 0.033)	0.074	−536.3
	Quartile 2	0.011	(−0.022, 0.044)	0.501	−536.5
	Quartile 3	−0.006	(−0.040, 0.028)	0.738	
	Quartile 4	0.036	(0.002, 0.071)	**0.041** *	
*Max CIMT* PM2.5	Linear	0.002	(0, 0.003)	**0.005** *	−477.4
	Log linear	0.030	(−0.005, 0.065)	0.081	−472.6
	Quartile 2	−0.008	(−0.045, 0.029)	0.674	−469.3
	Quartile 3	−0.001	(−0.039, 0.037)	0.949	
	Quartile 4	0.026	(−0.012, 0.063)	0.181	
BC	Linear	0.003	(−0.01, 0.02)	0.690	−389.7
	Log linear	−0.002	(−0.044, 0.041)	0.958	−389.6
	Quartile 2	0.007	(−0.036, 0.050)	0.746	−346.3
	Quartile 3	−0.014	(−0.057, 0.029)	0.522	
	Quartile 4	−0.016	(−0.058, 0.026)	0.448	
CO	Linear	0.040	(0, 0.08)	**0.048** *	−420.1
	Log linear	0.023	(0.002, 0.044)	**0.025** *	−421.2
	Quartile 2	0.017	(−0.022, 0.056)	0.395	−420.2
	Quartile 3	0.001	(−0.040, 0.042)	0.964	
	Quartile 4	0.05	(0.009, 0.092)	**0.018** *	

* Estimates for linear and log-linear models represent the change in mean CIMT (mm) per 10-unit increase in pollutant exposure (e.g., 10 μg/m^3^ for PM_2.5_ and BC, 10 ppm for CO). For quartile-based models, estimates represent the difference in mean CIMT (mm) for participants in quartiles 2, 3, and 4 of exposure compared to quartile 1 (reference group). Significant associations (p < 0.05) are bolded and indicated with an asterisk ().

## Data Availability

Data will be available upon request. To protect the confidentiality of study participants, identifiable information will be excluded from all datasets that will be made available. These will be accompanied by documentation necessary to understand the content (such as data dictionaries or metadata descriptions). All source datasets will be made available through the corresponding authors, subject to ethical, data protection, and other obligations being addressed. Data will also be published to a registry.

## Appendix A. Supplementary data

Supplementary data to this article can be found online at https://doi.org/10.1016/j.ijheh.2025.114649.
