# Supplementary material for: Household air pollution exposure in adult women is associated with increased carotid intima-media thickness: A cross-sectional study of the Household Air Pollution Intervention Network trial
Source: Int J Hyg Environ Health. Author manuscript; Available in PMC 2026 Jun 25. (PMC13295014; doi:10.1016/j.ijheh.2025.114649)
Supplement: Underhill_IJHEH_2025_SI [file NIHMS2165640-supplement-Underhill_IJHEH_2025_SI.docx]

**Household air pollution exposure in adult women is associated with increased carotid intima-media thickness: A cross-sectional study of the Household Air Pollution Intervention Network trial.**

[Supplemental Tables & Figures 2](#_Toc205475641)

[Figure S1. Directed acyclic graph representing the relationship between HAP exposure (green circle) and CIMT (blue circle). 2](#_Toc205475642)

[Figure S2. Correlation Matrix of mean CIMT and a priori HAP exposure factors and confounding factors 2](#_Toc205475643)

[Figure S3. Adjusted exposure-response relationships between 24-hour personal exposure to PM2.5, BC, and CO and carotid intima-media thickness (CIMT). 3](#_Toc205475644)

[Figure S4. Adjusted associations between quartiles of 24-hour personal air pollution exposure and carotid intima-media thickness (CIMT). 4](#_Toc205475645)

[Figure S5: GAM smooth function with 95% confidence intervals for a) PM2.5, b) BC, and c) CO. (d-f) Mean and 95% CIs of expected and observed CIMT by age. 5](#_Toc205475646)

[Figure S6. Distributions of personal exposure to PM_2.5_, BC, and CO in adult, non-pregnant women from the HAPIN trial. 5](#_Toc205475647)

[Figure S7. Unadjusted Correlations between (a) mean CIMT and (b) maximum CIMT and age, SBP, and personal PM_2.5_, BC, and CO exposure, by country. 6](#_Toc205475648)

[Table S1. Adjusted odds ratios for the association between personal 24-hour exposures—PM_2.5_ (µg/m^3^), BC (µg/^m3^), and CO (ppm)—and plaque presence. 6](#_Toc205475649)

# Supplemental Tables & Figures


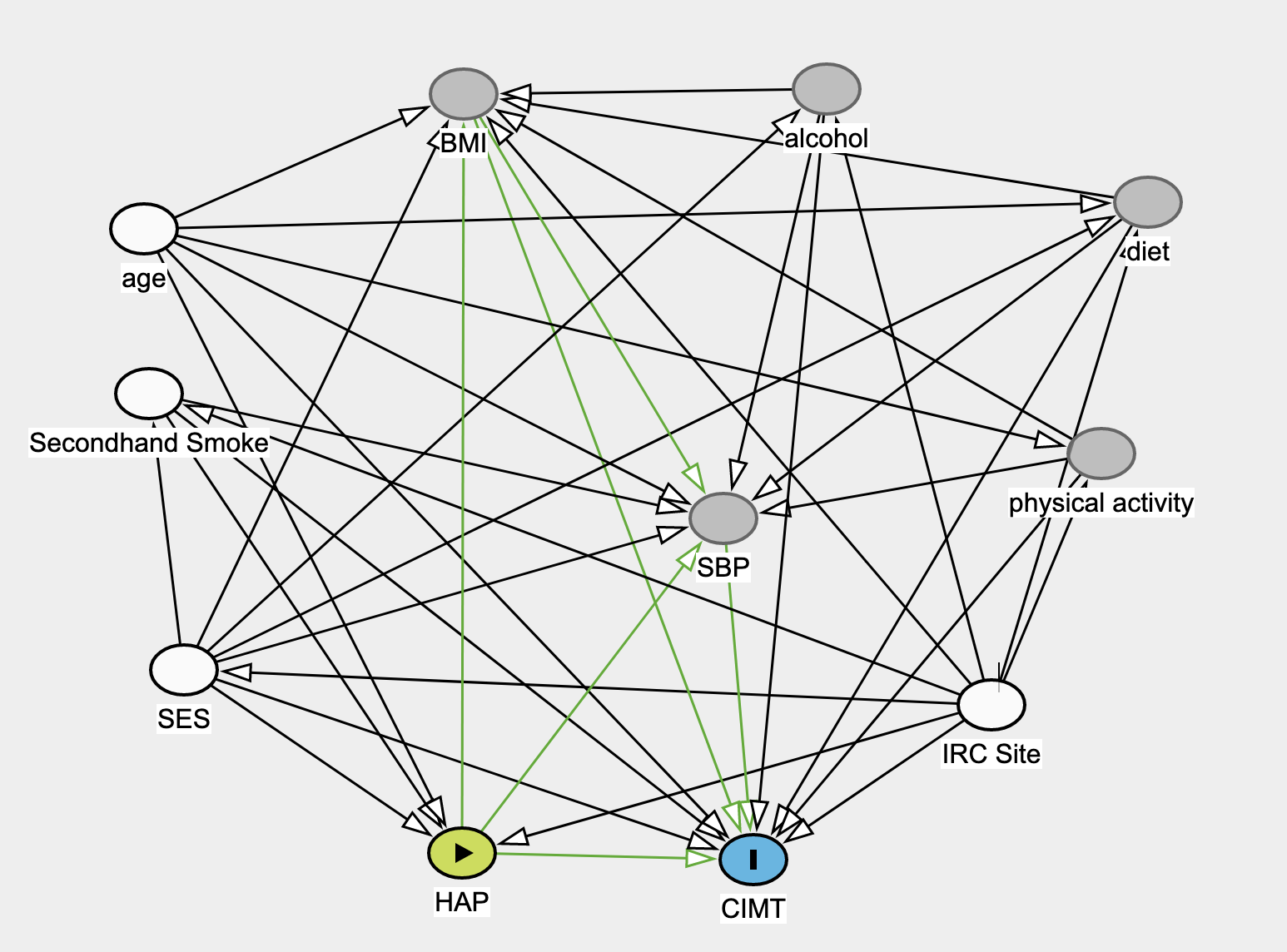


## Figure S1. Directed acyclic graph representing the relationship between HAP exposure (green circle) and CIMT (blue circle).

Hollow circles represent the *a priori* minimal sufficient adjustment set of factors used in all regression models. Green lines represent potential causal pathways.


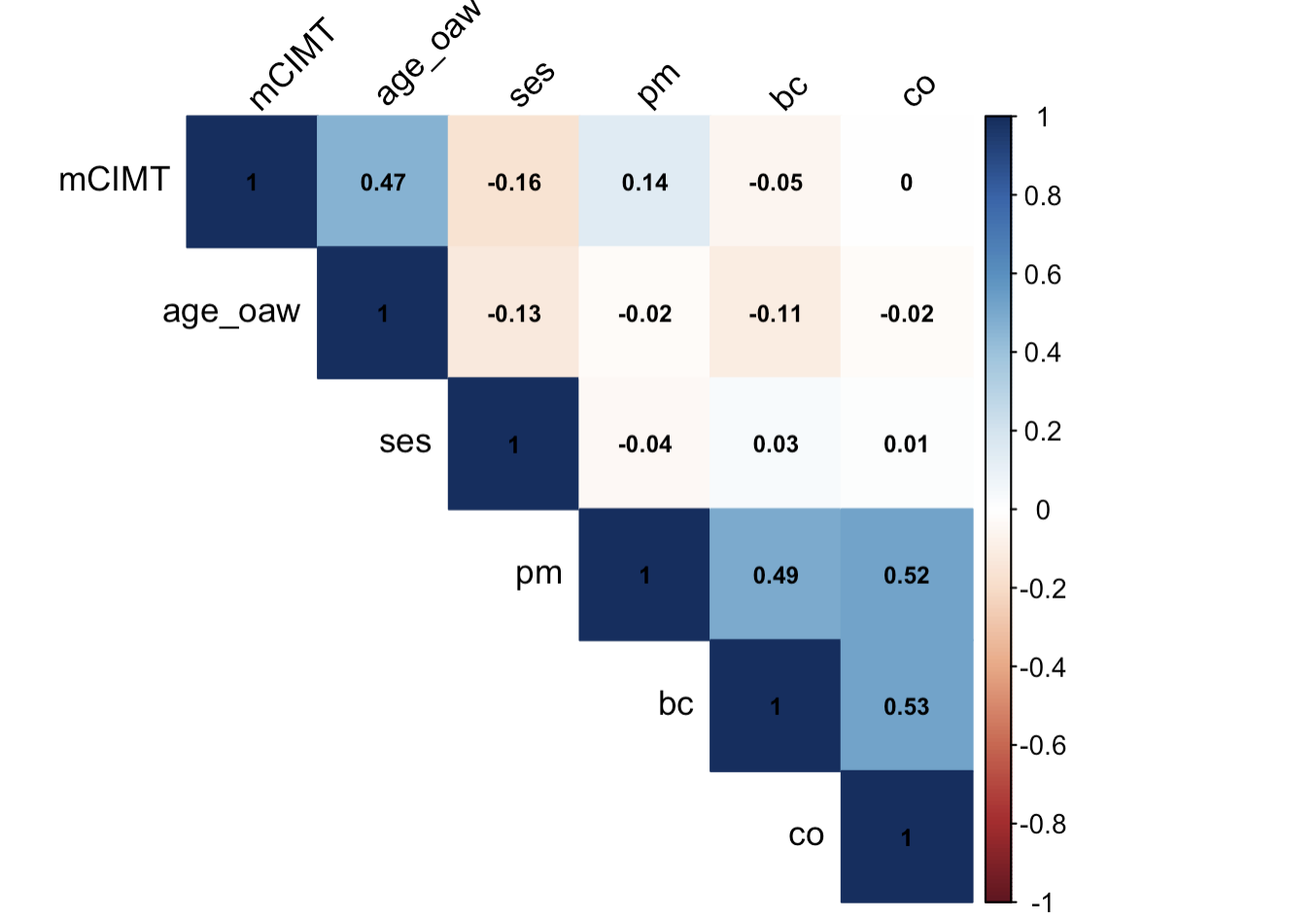


## Figure S2. Correlation Matrix of mean CIMT and a priori HAP exposure factors and confounding factors


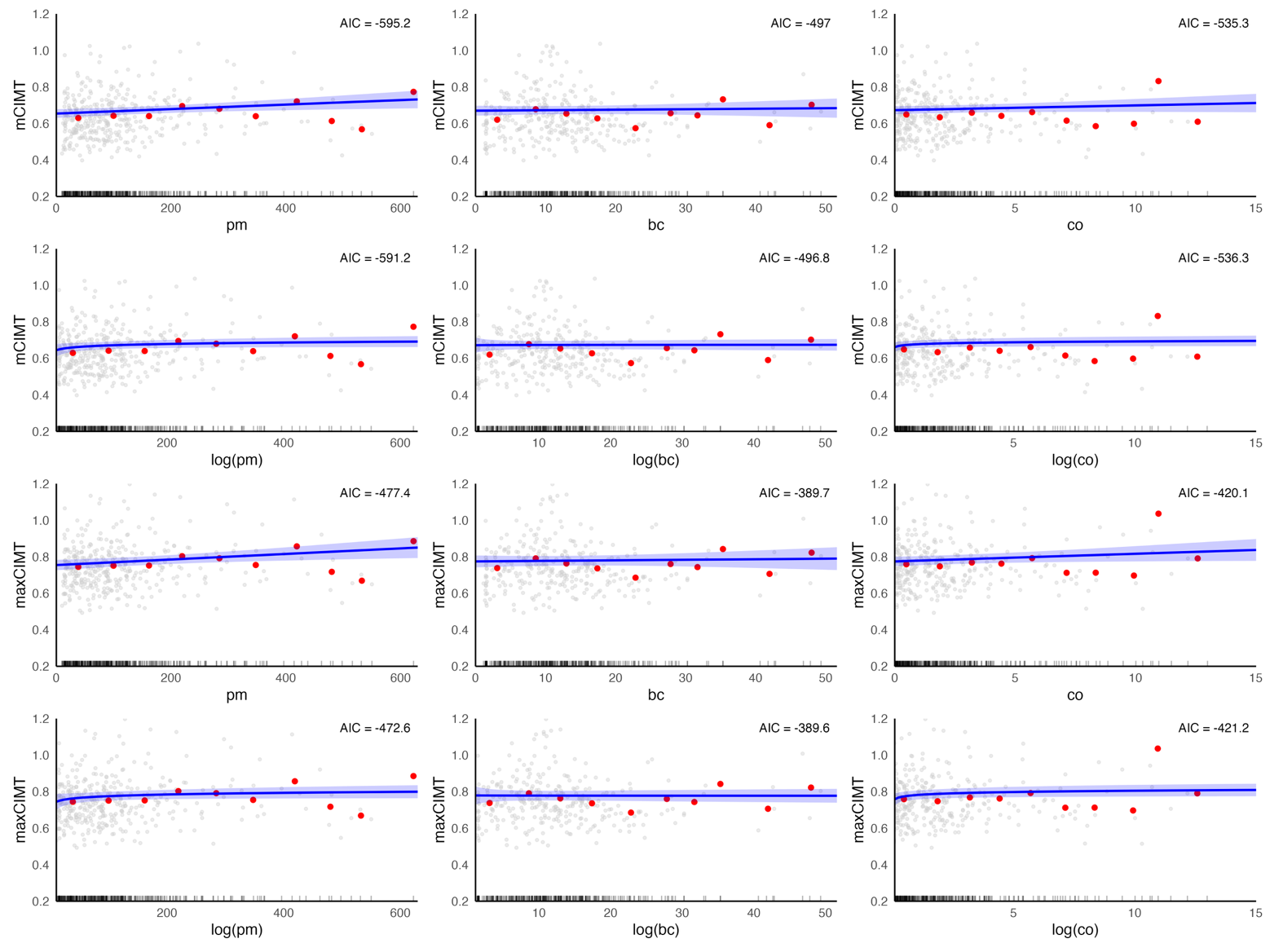


## Figure S3. Adjusted exposure-response relationships between 24-hour personal exposure to PM2.5, BC, and CO and carotid intima-media thickness (CIMT).

Each panel displays the relationship between a specific pollutant (PM₂.₅, black carbon [BC], or carbon monoxide [CO]) and either mean CIMT or maximum CIMT, modeled using linear or log-linear regression. Blue lines represent the adjusted model-predicted fit; shaded regions represent the 95% confidence interval. Red points show the average CIMT within binned exposure intervals. Gray dots represent individual observed values. AIC values are displayed in the top right of each plot as a measure of model fit (lower AIC indicates better fit). Models were adjusted for age, socioeconomic status, secondhand smoke exposure, and study site.


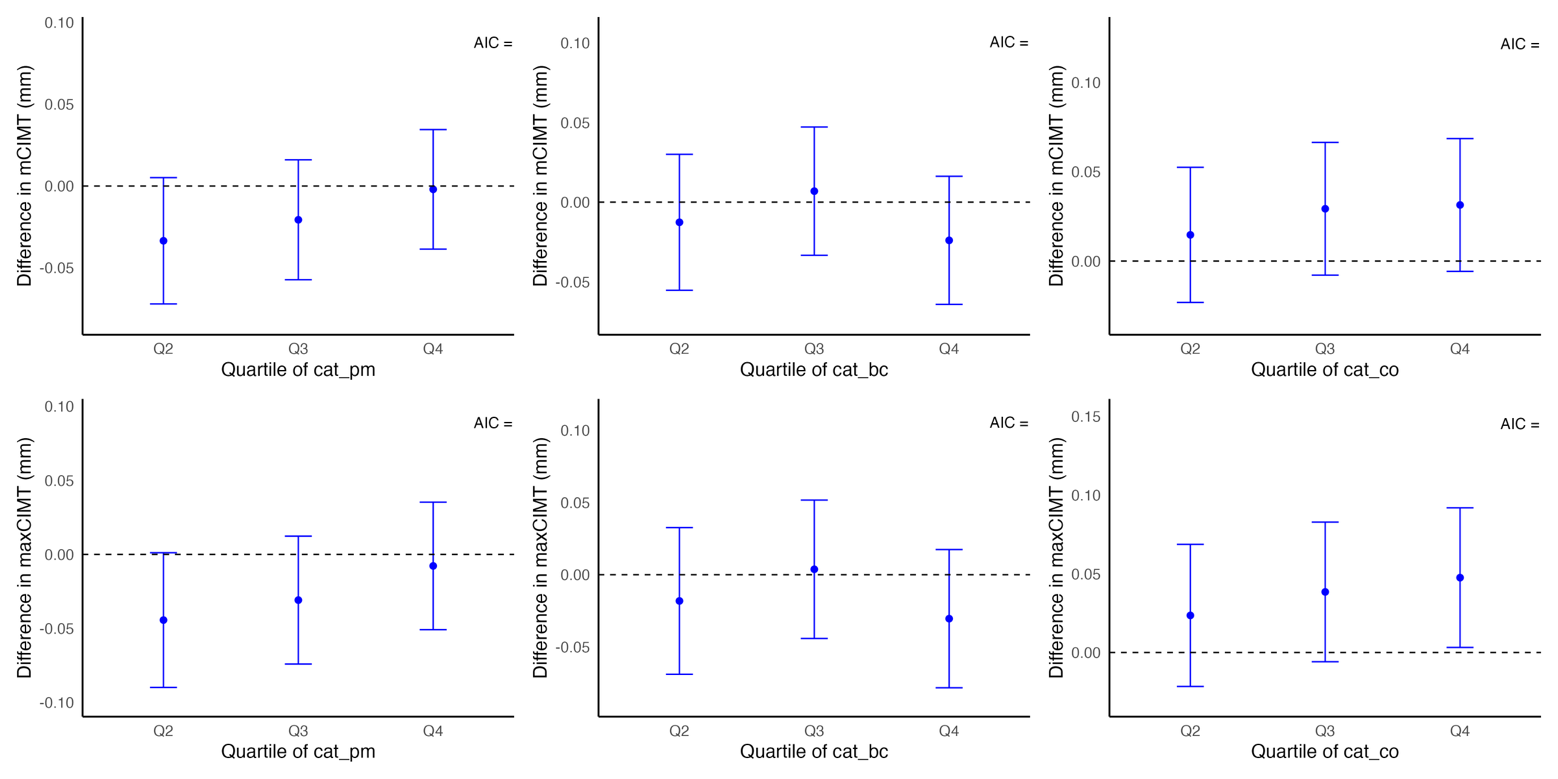


## Figure S4. Adjusted associations between quartiles of 24-hour personal air pollution exposure and carotid intima-media thickness (CIMT).

Adjusted differences in mean and maximum CIMT (mm) are shown by quartiles of 24-hour personal exposure to PM₂.₅, black carbon (BC), and carbon monoxide (CO). Quartile 1 (Q1) was used as the reference category. Models were adjusted for age, socioeconomic status, secondhand smoke exposure, and indoor residual spraying (IRC). Point estimates represent the mean difference in CIMT for each quartile compared to Q1, with 95% confidence intervals. AIC values are displayed for each model. Models were adjusted for age, socioeconomic status, secondhand smoke exposure, and study site.


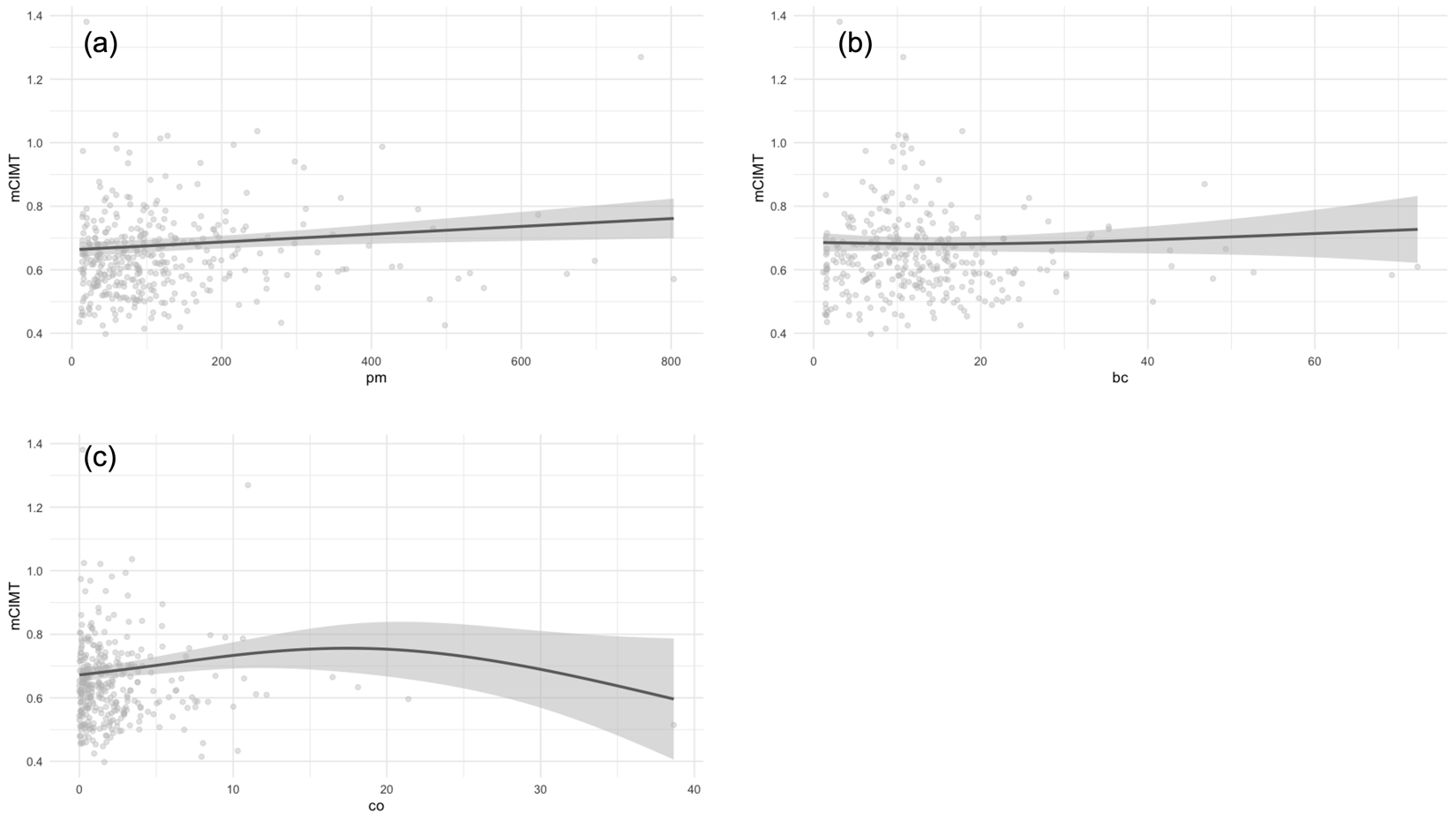


## Figure S5: GAM smooth function with 95% confidence intervals for a) PM2.5, b) BC, and c) CO. (d-f) Mean and 95% CIs of expected and observed CIMT by age.


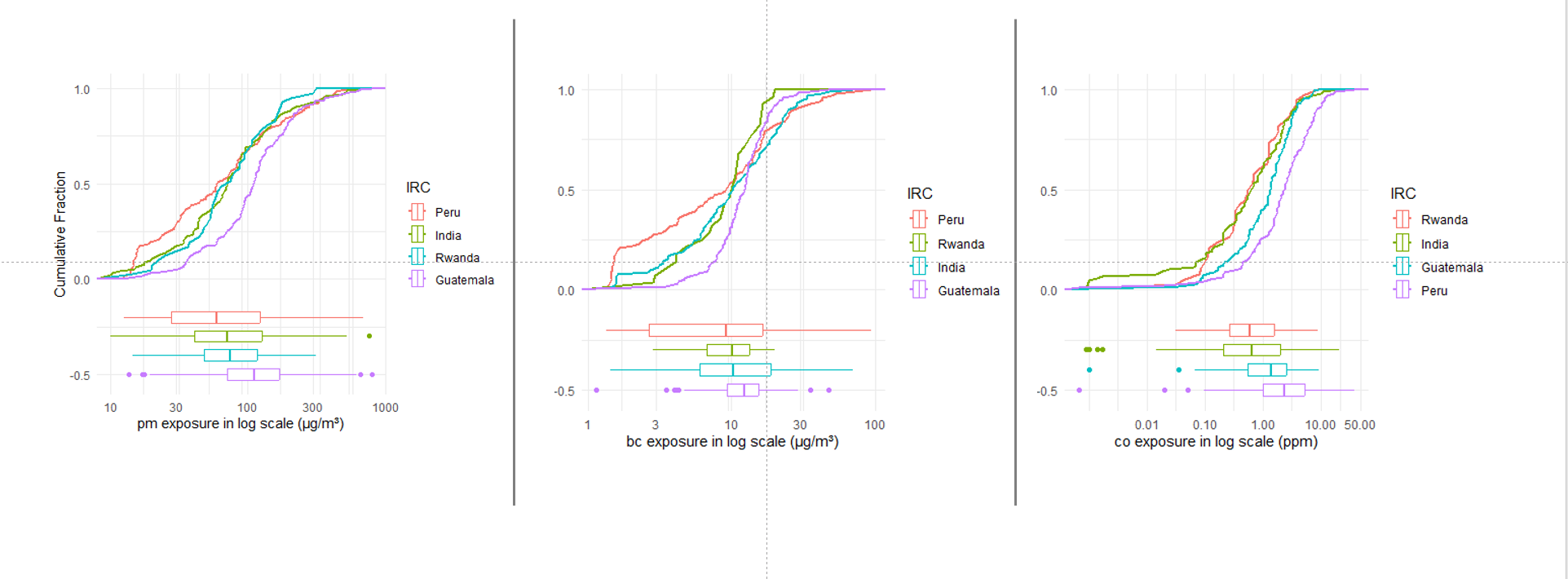


## Figure S6. Distributions of personal exposure to PM_2.5_, BC, and CO in adult, non-pregnant women from the HAPIN trial.

Site-specific exposure distributions were modeled using cumulative distribution functions and boxplots for all participants with valid exposure and CIMT measurements.


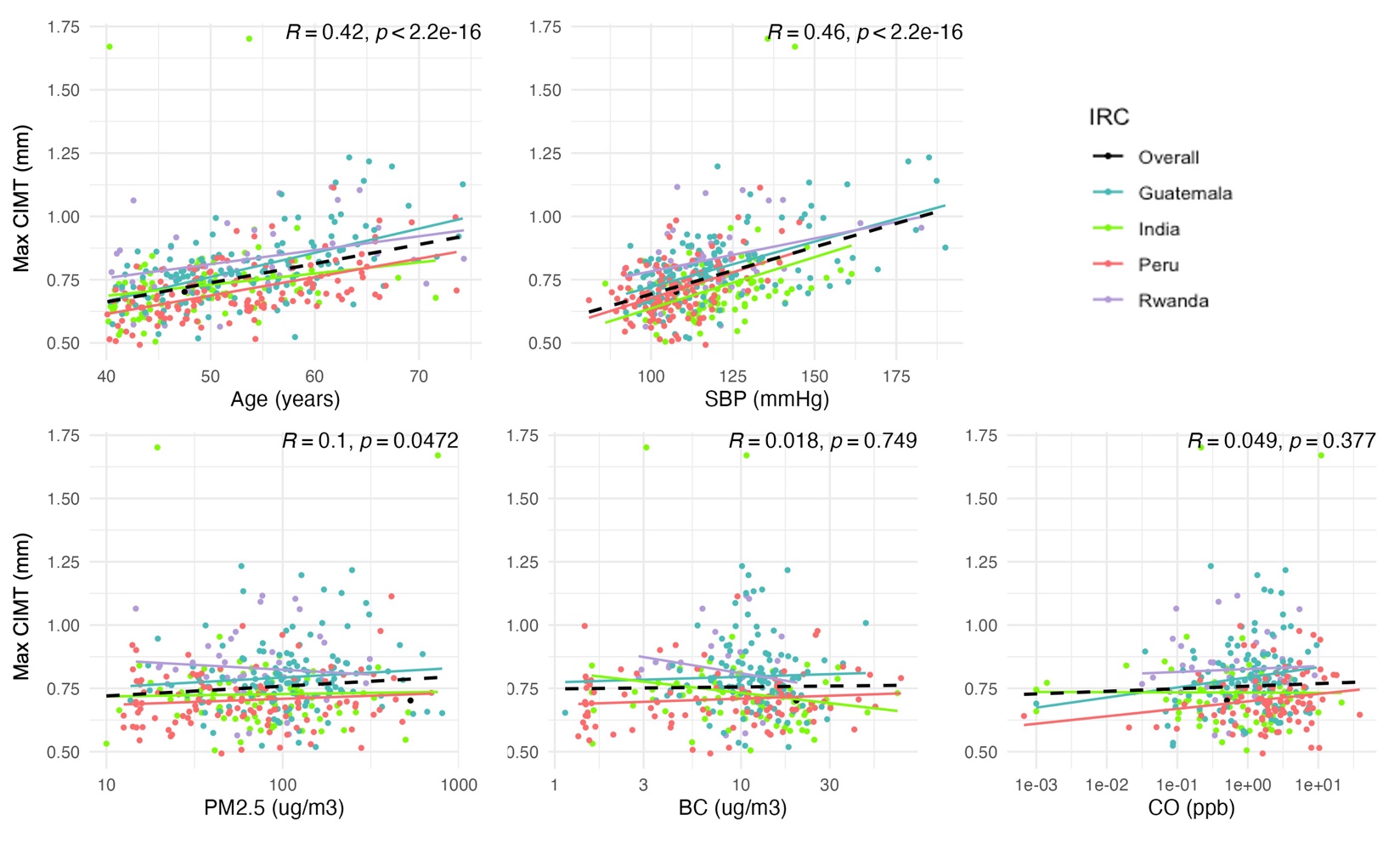


## Figure S7. Unadjusted Correlations between (a) mean CIMT and (b) maximum CIMT and age, SBP, and personal PM_2.5_, BC, and CO exposure, by country.

## Table S1. Adjusted odds ratios for the association between personal 24-hour exposures—PM_2.5_ (µg/m^3^), BC (µg/^m3^), and CO (ppm)—and plaque presence.

| **Pollutant** | **Model Type** | **Odds Ratio** | **95% CI** | **p-value** | **AIC** |
| --- | --- | --- | --- | --- | --- |
|  |  |  |  |  |  |
| PM2.5 | Linear | 1.00 | (1, 1) | 0.426 | 185.3 |
|  | Log linear | 1.17 | (0.71, 1.91) | 0.544 | 185.6 |
|  | Quartile 2 | 1.09 | (0.26, 5.6) | 0.909 | 189.7 |
|  | Quartile 3 | 1.27 | (0.33, 6.31) | 0.745 |  |
|  | Quartile 4 | 1.28 | (0.34, 6.26) | 0.729 |  |
| BC | Linear | 1.00 | (0.95, 1.05) | 0.892 | 173.9 |
|  | Log linear | 0.95 | (0.51, 1.81) | 0.862 | 173.9 |
|  | Quartile 2 | 0.28 | (0.06, 1.19) | 0.084 | 174.7 |
|  | Quartile 3 | 0.58 | (0.18, 2.1) | 0.382 |  |
|  | Quartile 4 | 0.43 | (0.12, 1.64) | 0.200 |  |
| CO | Linear | 1.08 | (0.94, 1.19) | 0.178 | 173.9 |
|  | Log linear | 0.87 | (0.69, 1.13) | 0.265 | 174.2 |
|  | Quartile 2 | 1.09 | (0.31, 4.41) | 0.900 | 175.9 |
|  | Quartile 3 | 0.37 | (0.08, 1.72) | 0.192 |  |
|  | Quartile 4 | 0.74 | (0.21, 3.04) | 0.656 |  |
